# Epidermal Growth Factor Receptor Activation in Glioblastoma through Novel Missense Mutations in the Extracellular Domain

**DOI:** 10.1371/journal.pmed.0030485

**Published:** 2006-12-19

**Authors:** Jeffrey C Lee, Igor Vivanco, Rameen Beroukhim, Julie H. Y Huang, Whei L Feng, Ralph M DeBiasi, Koji Yoshimoto, Jennifer C King, Phioanh Nghiemphu, Yuki Yuza, Qing Xu, Heidi Greulich, Roman K Thomas, J. Guillermo Paez, Timothy C Peck, David J Linhart, Karen A Glatt, Gad Getz, Robert Onofrio, Liuda Ziaugra, Ross L Levine, Stacey Gabriel, Tomohiro Kawaguchi, Keith O'Neill, Haumith Khan, Linda M Liau, Stanley F Nelson, P. Nagesh Rao, Paul Mischel, Russell O Pieper, Tim Cloughesy, Daniel J Leahy, William R Sellers, Charles L Sawyers, Matthew Meyerson, Ingo K Mellinghoff

**Affiliations:** 1 Department of Medical Oncology and Center for Cancer Genome Discovery, Dana-Farber Cancer Institute Harvard Medical School, Boston, Massachusetts, United States of America; 2 Department of Pathology, Harvard Medical School, Boston, Massachusetts, United States of America; 3 Broad Institute of Harvard and Massachusetts Institute of Technology, Cambridge, Massachusetts, United States of America; 4 Department of Molecular & Medical Pharmacology, David Geffen School of Medicine, University of California Los Angeles, Los Angeles, California, United States of America; 5 Department of Medicine, Harvard Medical School, Boston, Massachusetts, United States of America; 6 Department of Human Genetics, David Geffen School of Medicine, University of California Los Angeles, Los Angeles, California, United States of America; 7 Department of Pathology and Laboratory Medicine, David Geffen School of Medicine, University of California Los Angeles, Los Angeles, California, United States of America; 8 Department of Medicine, David Geffen School of Medicine, University of California Los Angeles, Los Angeles, California, United States of America; 9 Department of Neurology, David Geffen School of Medicine, University of California Los Angeles, Los Angeles, California, United States of America; 10 Department of Medicine, Brigham and Women's Hospital, Boston, Massachusetts, United States of America; 11 Department of Neurosurgery, University of California San Francisco, San Francisco, California, United States of America; 12 Department of Neurosurgery, David Geffen School of Medicine, University of California Los Angeles, Los Angeles, California, United States of America; 13 Department of Biophysics and Biophysical Chemistry, The Johns Hopkins University School of Medicine, Baltimore, Maryland, United States of America; 14 Howard Hughes Medical Institute, The Johns Hopkins University School of Medicine, Baltimore, Maryland, United States of America; 15 Howard Hughes Medical Institute, University of California Los Angeles, Los Angeles, California, United States of America; Memorial Sloan Kettering Cancer Center, United States of America

## Abstract

**Background:**

Protein tyrosine kinases are important regulators of cellular homeostasis with tightly controlled catalytic activity. Mutations in kinase-encoding genes can relieve the autoinhibitory constraints on kinase activity, can promote malignant transformation, and appear to be a major determinant of response to kinase inhibitor therapy. Missense mutations in the EGFR kinase domain, for example, have recently been identified in patients who showed clinical responses to EGFR kinase inhibitor therapy.

**Methods and Findings:**

Encouraged by the promising clinical activity of epidermal growth factor receptor (EGFR) kinase inhibitors in treating glioblastoma in humans, we have sequenced the complete *EGFR* coding sequence in glioma tumor samples and cell lines. We identified novel missense mutations in the extracellular domain of *EGFR* in 13.6% (18/132) of glioblastomas and 12.5% (1/8) of glioblastoma cell lines. These *EGFR* mutations were associated with increased *EGFR* gene dosage and conferred anchorage-independent growth and tumorigenicity to NIH-3T3 cells. Cells transformed by expression of these *EGFR* mutants were sensitive to small-molecule EGFR kinase inhibitors.

**Conclusions:**

Our results suggest extracellular missense mutations as a novel mechanism for oncogenic EGFR activation and may help identify patients who can benefit from EGFR kinase inhibitors for treatment of glioblastoma.

## Abstract 2

Ingo Mellinghoff and colleagues sequenced the*EGFR* gene in glioblastoma samples and cell lines and identified missense mutations in the extracellular domain that suggest a new mechanism for EGFR activation.

## Editors' Summary

**Background.:**

Normally, cell division (which produces new cells) and cell death are finely balanced to keep the tissues and organs of the human body in working order. But sometimes, cells acquire changes (mutations) in their genetic material that allow them to divide uncontrollably to form cancers—life-threatening, disorganized masses of cells. Cancer treatments often involve drugs that kill rapidly dividing cells but, although these hit cancer cells hardest, they also damage some normal tissues. Now, though, some of the specific changes that allow cancer cells to divide uncontrollably have been identified and drugs that attack only these abnormal cells are being developed. One of these—erlotinib—inhibits the activity of epidermal growth factor receptor (EGFR), a “receptor tyrosine kinase” that sits in the cell membrane. The interaction of epidermal growth factor (EGF)—a messenger protein—with the extracellular portion (or domain) of EGFR activates its intracellular part (a kinase enzyme). This adds phosphate groups to tyrosine (an amino acid) in proteins that form part of a signaling cascade that tells cells to divide. Cancer cells often have alterations in EGFR signaling. Some have extra copies of the *EGFR* gene (*EGFR* amplification); others make a short version of EGFR that is always active because it lacks the extracellular domain that binds EGF; yet others contain EGFR that is permanently active because of mutations in its kinase domain.

**Why Was This Study Done?:**

Erlotinib can help only patients whose tumor growth is dependent on EGFR signaling. To identify these patients it is necessary to have a detailed catalog of the mutations that occur in EGFR in tumors and to know which mutations drive uncontrolled cell growth. In this study, the researchers have catalogued and characterized the mutations in EGFR that occur in glioblastoma, a deadly type of brain tumor. The researchers chose this tumor type for their study because *EGFR* amplification and loss of the extracellular domain of EGFR are both common in glioblastomas and because about one in five patients with glioblastoma responds well to EGFR kinase inhibitors.

**What Did the Researchers Do and Find?:**

The researchers sequenced the whole coding sequence of the *EGFR* gene in more than 100 glioblastomas. Nearly 15% of the tumors contained missense mutations—changes that alter the amino acid sequence of EGFR. Only one tumor had a mutation in the EGFR kinase domain; the rest had mutations in its extracellular domain. To test whether these newly identified mutations might contribute to cancer development (oncogenesis), the researchers introduced mutated or normal *EGFR* genes into nontumorigenic mouse cells. Only the cells that contained the mutated *EGFR* genes formed tumors when injected into mice, indicating that the nontumorigenic cells had been “transformed” into cancer cells by the mutated *EGFR* genes. Finally, the researchers showed that EGFR containing the extracellular missense mutations had kinase activity in the absence of EGF when expressed in human and mouse cells, and that the growth of cells transformed by expression of the mutated genes was sensitive to erlotinib.

**What Do These Findings Mean?:**

These findings identify missense mutations in the extracellular domain of EGFR as a new way to oncogenically activate this protein. Until now researchers have concentrated on the kinase domain of this and other receptor tyrosine kinases in their search for oncogenic mutations, but the results of this study suggest that future searches should be much broader. The distribution of EGFR missense mutations in glioblastoma contrasts with that in lung cancer, in which alterations in EGFR signaling are also implicated in cancer development but all the oncogenic mutations are in the kinase domain. Fortunately, EGFR kinase inhibitors like erlotinib have broad activity: They inhibit the growth of cells transformed by the expression of EGFR containing extracellular domain mutations or kinase mutations, or by the expression of the short EGFR variant. This bodes well for the use of these drugs in patients with glioblastoma. However, before these inhibitors become a standard part of cancer treatments, sensitive techniques need to be developed to analyze tumors for these mutations so that the patients who will benefit from these targeted therapies can be identified.

**Additional Information.:**

Please access these Web sites via the online version of this summary at http://dx.doi.org/10.1371/journal.pmed.0030485.

- MedlinePlus encyclopedia entries on cancer and on brain tumors
- US National Cancer Institute information for patients and professionals on brain tumors
- Wikipedia pages on protein kinases, epidermal growth factor receptor, and erlotinib (note that Wikipedia is a free online encyclopedia that anyone can edit)

## Introduction

The epidermal growth factor receptor (EGFR) is a receptor tyrosine kinase that regulates fundamental processes of cell growth and differentiation. Deletion of the *EGFR* gene is embryonically lethal in mice, and increased EGFR signaling has been linked to a variety of human malignancies. Mechanisms for oncogenic conversion of EGFR in cancer include *EGFR* gene amplification, structural rearrangements of the receptor, overexpression of epidermal growth factor (EGF)–family ligands by tumor cells and/or surrounding stroma, and—as was recently shown in lung cancer—activating mutations in the *EGFR* kinase domain [1].

The evidence for a role of EGFR in oncogenesis is particularly compelling in glioblastoma, the most aggressive human brain tumor with a two year survival of less than 5% despite surgery, radiation, and chemotherapy [2,3]. About 40% of glioblastomas show amplification of the *EGFR* gene locus [4], and about half of these tumors express a mutant receptor (EGFRvIII) that is constitutively active due to an in-frame truncation within the extracellular ligand-binding domain [5–7]. Perhaps the strongest evidence for a role of EGFR in the biology of glioblastoma stems from clinical trials in which 15%–20% of glioblastoma patients experienced significant tumor regression in response to small-molecule EGFR kinase inhibitors [8,9]. Our recent data indicate that expression of EGFRvIII in the context of an intact PTEN (phosphatase and tensin homolog) pathway is associated with these clinical responses [9].

To explore the possibility that *EGFR* might be the target of oncogenic mutations outside the kinase domain, we sequenced the entire *EGFR* coding region in a panel of 151 glioma tumors and cell lines.

## Methods

### DNA Samples

Genomic DNA was extracted from eight glioblastoma cell lines (A172, SF268, SF295, SF539, T98G, U87, U118, and U251) and 143 fresh frozen glioma samples. The clinical glioma samples comprised glioblastomas (*n* = 132), World Health Organization grade III anaplastic astrocytomas (*n* = 3), grade III mixed gliomas (*n* = 4), and grade III oligodendrogliomas (*n* = 4). Germline genomic DNA was extracted from peripheral blood samples. To confirm the match between germline and tumor DNA for each patient, we performed mass spectrometric genotyping of 24 single-nucleotide polymorphism (SNP) loci. These loci included 23 SNP loci represented on both 50K Xba and Hind arrays (Affymetrix, http://www.affymetrix.com) and one AmelXY locus for sex determination (Table S1). Collection and analysis of all clinical samples was approved by the University of California Los Angeles Institutional Review Board.

### Reagents

Erlotinib was purchased from WuXi Pharmatech (http://www.pharmatechs.com). The following antibodies were used in this study: anti-EGFR, anti-phospho-Y1068-EGFR anti-phospho-Y845-EGFR, and anti-phosphoinositide 3-kinase (PI3K) p85 (all from Cell Signaling Technology, http://www.cellsignal.com); anti-phosphotyrosine 4G10 (Upstate Biotechnologies, now Millipore, http://www.upstate.com); and anti-actin, anti-ERK1/2, and anti-P-ERK1/2 (all from Santa Cruz Biotechnology, http://www.scbt.com).

### Sequencing and Mass Spectrometric Genotyping

PCR reactions for each exon and flanking intronic sequences contained 5 ng of genomic DNA, 1× HotStar Buffer, 0.8 mM dNTPs, 1 mM MgCl_2_, 0.2 U HotStar Enzyme (Qiagen, http://www.qiagen.com), and 0.2 μM forward and reverse primers in a 6 or 10 μl reaction volume. PCR cycling parameters were: one cycle of 95 °C for 15 min; 35 cycles of 95 °C for 20 s, 60 °C for 30 s, and 72 °C for 1 min; followed by one cycle of 72 °C for 3 min.

The resulting PCR products were sequenced using bidirectional dye-terminator fluorescent sequencing with universal M13 primers. Sequencing fragments were detected via capillary electrophoresis using ABI Prism 3730 DNA Analyzer (Applied Biosystems, http://www.appliedbiosystems.com). PCR and sequencing were performed at Agencourt Bioscience Corporation (http://www.agencourt.com) or at the Broad Institute of Harvard and MIT (http://www.broad.mit.edu). Forward (F) and reverse (R) chromatograms were analyzed in batch with Mutation Surveyor 2.51 (SoftGenetics, http://www.softgenetics.com), followed by manual review.

A minimum of 21 of 28 (75%) *EGFR* exon sequence coverage was accomplished for 151 samples. An exon for each individual sample was considered covered if 90% of the sequence trace within the exon had a phred quality score of 30 or greater, a signal-to-background noise ration of 15% or less, and signal intensity greater than 25% of the signal intensity of the sequencing plate. High-quality sequence variations found in one or both directions were scored as candidate mutations. Exons harboring candidate mutations were reamplified from the original DNA sample and resequenced.

For mass spectrometric genotyping, PCR and extension primers (Table S2) were designed using SpectroDESIGNER software (Sequenom, http://www.sequenom.com). Unincorporated nucleotides from PCR reactions were dephosphorylated with shrimp alkaline phosphatase (Amersham, http://www.amersham.com) followed by primer extension with ThermoSequence polymerase (Amersham). Primer extension reactions were loaded onto SpectroCHIPs (Sequenom) and analyzed using a MALDI-TOF (matrix-assisted laser desorption/ionization time-of-flight) mass spectrometer (SpectroREADER, Sequenom) [10]. Mass spectra were processed with SpectroTYPER (Sequenom) to determine genotypes based on peaks intensities corresponding to the expected extension products.

### Affymetrix 100K SNP Arrays

Genomic DNA was processed and hybridized following the guidelines of the manufacturer (Affymetrix) and arrays were scanned with a GeneChip Scanner 3000. Genotyping calls and signal quantification were obtained using GeneChip Operating System 1.1.1 and Affymetrix Genotyping Tools 2.0 software. Data were normalized at the probe level to a baseline array with median signal intensity using invariant set normalization. After normalization, the signal values for each SNP in each array were obtained with a model-based (perfect-match/mismatch) method [11]. Signal intensities at each probe locus were compared with a set of normal reference samples representing 36 ethnically matched individuals to generate log_2_ ratios. Log_2_ ratios were smoothed using the breakpoint analysis method in the R package GLAD (Gain and Loss Analysis of DNA) [12]. Regions were considered amplified if their smoothed log_2_ ratio exceeded 0.3 (half the variation seen with a single-copy gain).

### Fluorescence In Situ Hybridization

Dual-probe fluorescence in situ hybridization (FISH) was performed on paraffin-embedded sections with locus-specific probes for *EGFR* and the centromere of Chromosome 7 as previously described [9].

### Determination of *EGFRvIII* Expression

RNA was extracted from fresh frozen tumor samples and *EGFRvIII* expression determined by two independent RT-PCR assays for each sample. Primer pairs included: #1F 5′-CTTCGGGGAGCAGCGATGCGAC-3′, #1R 5′-ACCAATACCTATTCCGTTACAC-3′, #2F 5′-GAGCTCTTCGGGGAGCAG-3′, and #2R 5′-GTGATCTGTCACCACATAATTACCTTTCTT-3′. EGFRvIII expression was also examined by immunohistochemistry and/or immunoblotting depending on the availability of tissue samples.

### Quantification of Mutant *EGFR* Alleles

The abundance of missense and wild-type *EGFR* alleles in tumor DNA samples was determined by PCR-cloning and sequencing of respective *EGFR* exons. PCR products were ligated into PCR2.1-Topo vectors (Invitrogen) and transformed into *E. coli.* After transformation, bacteria were plated onto selection plates and grown overnight. For each DNA sample, 65–94 colonies were isolated using a colony picking robot (QPix2, Genetix Limited, http://www.genetix.com), grown overnight, and bidirectionally sequenced at the Broad Institute. Sequence traces were analyzed using Mutation Surveyor software (SoftGenetics).

### *EGFR* Expression Constructs

Retroviral *EGFR* expression constructs containing puromycin (pBabe-puro-*EGFR*) [13] or neomycin-resistance genes (pLXSN-neo-*EGFR*) [14] were used for site-directed mutagenesis using the Quick-Change Mutagenesis XL kit (Stratagene, http://www.stratagene.com). pLXSN-neo-*EGFR* retroviral constructs for EGFR and EGFRvIII were generously provided by David Riese 2nd (Purdue University, West Lafayette, Illinois, United States) and Webster Cavenee (Ludwig Institute for Cancer Research, La Jolla, California, United States). pBabe-Puro-based viral stocks were generated by transfecting the Phoenix 293T packaging cell line (Orbigen, http://www.orbigen.com) with the pBabe-Puro retroviral constructs using Lipofectamine 2000 (Invitrogen, http://www.invitrogen.com). pLXSN-Neo-based viral stocks were generated by transfecting the human amphotrophic 293-T cell line with pLXSN-Neo retroviral constructs using Lipofectamine 2000 (Invitrogen). Supernatants were collected 24–48 h post-transfection, filtered (0.45 μM), and used to infect NIH-3T3 cells, Ba/F3 cells, and human astrocytes.

### Expression of *EGFR* Alleles in NIH-3T3 Cells

Cells cultured in DMEM supplemented with 10% calf serum were infected with pBabe-Puro-based viral stock in the presence of polybrene. Beginning 2 d after infection, cells were selected in puromycin (2 μg/ml) for 3 d. Pooled NIH-3T3 cells stably expressing respective *EGFR* alleles at comparable EGFR protein levels were examined for their ability to induce colony formation in soft agar and tumor growth in nude mice. For soft agar assays, 1 × 10^5^ NIH-3T3 cells were suspended in a top layer of DMEM supplemented with 10% calf serum and 0.4% Select Agar (Gibco/Invitrogen) and plated on a bottom layer of DMEM supplemented with 10% calf serum and 0.5% Select Agar. EGF (10 ng/ml) was added to the top agar where indicated. Pictures of colonies were taken 2–3 wk after plating. Colonies were counted from ten random images (40× magnification) taken from each well. Colonies were counted from three replicate wells with the average number represented. In vivo tumorigenicity assays were performed in three mice (two injections/mouse) for each cell line. For each injection, 2 × 10^6^ cells were injected subcutaneously into each nude mouse (Taconic, http://www.taconic.com) and three-dimensional tumor volumes calculated 3–4 wk following injection.

### Expression of *EGFR* Alleles in Ba/F3 Cells

Murine Ba/F3 pro-B lymphocytes [15] were cultured in RPMI 1640 (Cellgro, Mediatech, http://www.cellgro.com) supplemented with 10% FCS, 100 units/ml penicillin and 100 μg/ml streptomycin, 1% L-glutamine, and 10% WEHI-3B-conditioned media. To derive Ba/F3 subclones stably expressing various *EGFR* alleles, Ba/F3 cells were “spinfected” with pBabe-puro-*EGFR*-based viral supernatants and spinfection repeated after 48 h. Cells were selected for neomycin or puromycin resistance and maintained in the presence of interleukin-3 (IL-3). IL-3-independent subclones were derived through prolonged passage in IL-3-depleted media. To determine sensitivity to erlotinib, 1 × 10^3^ cells were seeded in 96-well flat-bottomed plates with the indicated concentrations of erlotinib. Cell proliferation was assessed 48 h postplating using the WST-1 assay (Roche, http://www.roche.com). Each data point represents the median of six replicate wells for each Ba/F3 subclone and erlotinib concentration.

### Expression of *EGFR* Alleles in Human Astrocytes

Viral supernatants (pLXSN-neo-*EGFR*) were used to infect immortalized human astrocytes expressing the catalytic subunit of the telomerase holoenzyme and human papillomavirus 16 E6/E7 [16]. Astrocytes were then selected in G418 (Invitrogen) for approximately 10 d.

## Results

### Missense Mutations in Glioblastoma Cluster in the Extracellular Domain of *EGFR*

Encouraged by the recent success in identifying oncogenic kinase mutations through resequencing of kinase-encoding genes [17–19], we sequenced the entire coding sequence of *EGFR* in 143 human glioma samples and eight glioblastoma cell lines. Analysis of the initial Sanger sequencing results in these 151 samples revealed several novel sequence variations in the coding region of the *EGFR*. To validate these candidate mutations via a complementary method, all DNA samples were reexamined using allele-specific genotyping by MALDI-TOF mass spectrometry.

**Figure 1:**
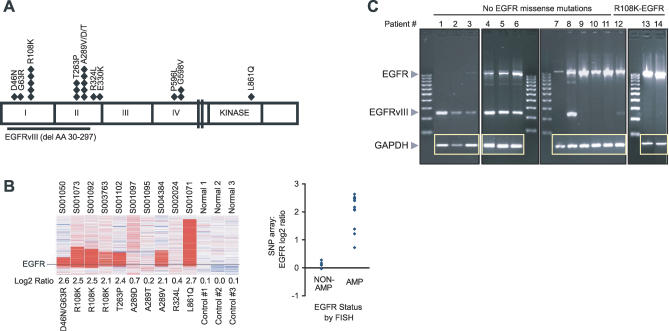
EGFR Missense Mutations in Glioblastoma Cluster in the Extracellular Domain and Are Associated with Increased *EGFR* Gene Dose (A) Location of missense mutations within the EGFR protein in a panel of 151 gliomas (132 glioblastomas, 11 WHO grade III gliomas, and eight glioblastoma cell lines). Each diamond represents one sample harboring the indicated mutation. Amino acid (AA) numbers are based on the new convention for EGFR numbering, which starts at the initiator methionine of pro-EGFR. Ligand-binding domains (I and III), cysteine-rich domains (II and IV), kinase domain (kinase), and the extracellular deletion mutant EGFRvIII [45] are indicated as reference. (B) Increased *EGFR* gene dose in tumors harboring *EGFR* missense mutations. The array (left) shows a high-resolution view of Affymetrix 100K SNP array at the *EGFR* gene locus for ten glioblastoma tumors and three normal controls (sample numbers are indicated above each column). *EGFR* mutation and log_2_ ratio (see Methods) are indicated below each column. The plot (left) shows a comparison of *EGFR* gene copy number determination by SNP array (y-axis, EGFR log_2_ ratios) and FISH (x-axis). AMP, amplified; NON-AMP, non amplified. (C) RT-PCR for *EGFRvIII* and full-length *EGFR* in 14 fresh-frozen glioblastoma tumors (see Methods). The upper band represents full-length *EGFR* (1,044 bp), the lower band *EGFRvIII* (243 bp), and the inset shows glyceraldehyde-3-phosphate dehydrogenase *(GAPDH)* RT-PCR results.

In all, we identified *EGFR* missense mutations in 14.4% (19/132) of glioblastomas, 12.5% (1/8) of glioblastoma cell lines, and none (0/11) in lower-grade gliomas. Only one tumor sample harbored a missense mutation in the *EGFR* kinase domain (L861Q), the location of *EGFR* mutations in lung cancer, supporting the recent conclusion from other groups that *EGFR* kinase domain mutations appear to be a rare event in this disease [20–22]. The remainder of the *EGFR* mutations (18/132 glioblastomas) were located in the extracellular ligand-binding (I, III) or cysteine-rich (II, IV) domains of the receptor (Figure 1A). Two evolutionarily highly conserved amino acid residues (Figure S1) were affected by mutations in five samples each (R108 and A289). Examination of peripheral blood DNA, matched to the tumor DNA by genotyping of 24 SNP loci, showed that eight of the 12 distinct missense mutations were unambiguously somatic, and one mutation (E330K) was germline. Three additional missense mutations (A289D, A289T, and R324L) were found in tumors for which no normal tissue was available (Table 1). None of the missense mutations were detected in germline DNA from 270 normal control individuals.

**Table 1:**
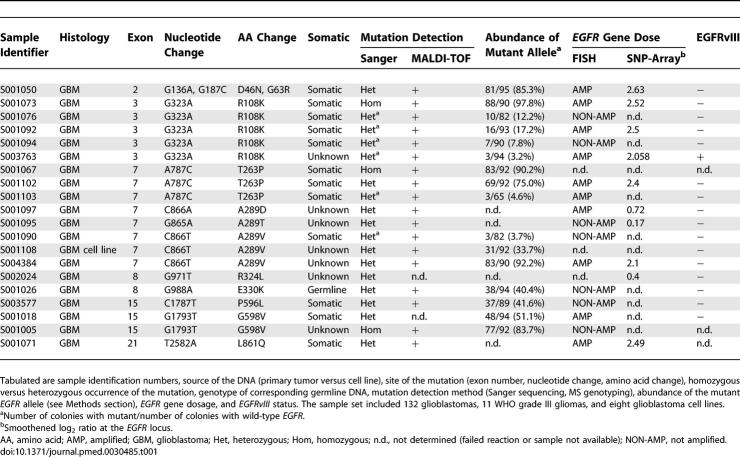
*EGFR* Missense Mutations Identified in a Panel of 151 Glioma Samples

To define which fraction of the *EGFR* pool represented the mutant allele in gliomas with *EGFR* missense mutations, we employed a PCR-cloning strategy previously used by our laboratories for mutation detection in clinical samples [23]. The mutant *EGFR* allele represented 30%–98% of the receptor pool in two-thirds (10/16) of all examined cases and over 50% in at least one tumor representing the most common amino acid changes: R108K, T263P, A289V, and G598V (Table 1). Lower abundance of the mutant *EGFR* allele in other samples might be due to contaminating stromal tissue, because genomic DNA was extracted from frozen tumor aliquots without prior microdissection.

We also genotyped genomic DNA from 119 primary lung tumors to detect *EGFR* ectodomain mutations. While 13.4% (16/119) of these lung tumor samples harbored mutations in the *EGFR* kinase domain, we did not detect any of the glioma-related *EGFR* ectodomain mutations in this sample set.

### *EGFR* Ectodomain Mutations Are Associated with Increased *EGFR* Gene Dose

Since *EGFR* is amplified in about 40% of human glioblastomas [4], we determined the relationship between *EGFR* missense mutation and *EGFR* gene dose in our tumor samples. Of 17 tumors with *EGFR* missense mutations, 58.8% (10) showed evidence for *EGFR* amplification by FISH and/or Affymetrix 100K SNP genotyping arrays (Figure 1B; Table 1). This distribution suggests that *EGFR* missense mutations are associated with *EGFR* amplification and raises the question of whether *EGFR* missense mutations in glioblastoma co-occur with or are mutually exclusive of the *EGFRvIII* mutation, which is found almost exclusively in glioblastomas with increased gene dosage [24]. Using at least two independent assays for the determination of *EGFRvIII* status, we identified the *EGFRvIII* allele in 28.3% (13/46) of gliomas without *EGFR* missense mutation and 6.3% (1/16) tumors with *EGFR* missense mutation (Figure 1C; Table 1); note that this tumor showed vastly lower levels of *EGFRvIII* (Figure 1C, lane 12). These findings suggest that *EGFR* ectodomain mutations occur independently of *EGFRvIII* in glioblastoma and provide an alternative mechanism for EGFR activation in this disease.

### *EGFR* Ectodomain Mutants Are Oncogenic

**Figure 2:**
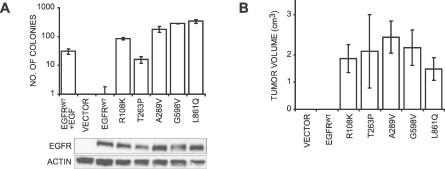
EGFR Missense Mutations Are Transforming and Tumorigenic (A) Anchorage-independent growth of NIH-3T3 cells expressing various *EGFR* alleles as mean number of colonies ± standard deviation (bar graph, above). The lanes (below) show EGFR and actin immunoblots of whole cell lysates from NIH-3T3 subclones plated in soft agar. EGF (10 ng/ml) was added to the top agar where indicated. (B) Tumorigenicity of NIH-3T3 cells stably expressing the indicated *EGFR* alleles in nude mice. Mean tumor size ± standard deviation was determined 3–4 wk after subcutaneous inoculation into nude mice (*n* = 6 per cell line).

To test the oncogenicity of the glioma-related *EGFR* missense mutations, we transduced NIH-3T3 fibroblasts with retroviruses encoding either wild-type *EGFR* or selected *EGFR* missense mutants (encoding R108K, T263P, A289V, G598V, and L861Q). Ectopic expression of all *EGFR* mutants examined in NIH-3T3 cells conferred anchorage-independent colony formation in soft agar (Figure 2A). In contrast, expression of wild-type *EGFR* induced a transformed phenotype only in the presence of exogenous EGF, as previously reported [25,26].

To further analyze the oncogenic potential of the *EGFR* mutants, NIH-3T3 subclones stably expressing the same missense mutant receptors (encoding R108K, T263P, A289V, G598V, and L861Q) were inoculated subcutaneously into nude mice. NIH-3T3 cells infected with empty vector or wild-type *EGFR*-expressing virus did not yield any measurable tumors within the four-week observation period. In contrast, NIH-3T3 cells expressing each of the tested *EGFR* missense mutants produced large tumors at the inoculation site in all mice within three to four weeks (Figure 2B).

### EGFR Ectodomain Mutants Are Basally Phosphorylated and Are Responsive to Ligand

**Figure 3:**
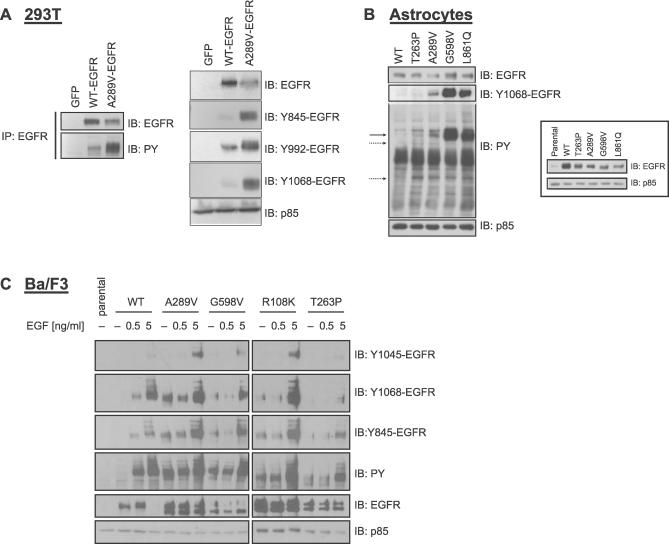
Basal Activation and Ligand Response of EGFR Ectodomain Mutants (A) Increased EGFR tyrosine phosphorylation of A289V-EGFR. 293T cells were transiently transfected with green fluorescent protein (GFP; control), wild-type *EGFR*, or A289V-*EGFR*. At 24 h after transfection, 24 cells were serum starved for 12 h and then lysed. Shown are immunoblots of immunoprecipitated EGFR (left blots) and whole cell lysates (right blots). (B) Increased basal activity of EGFR missense mutants in human astrocytes. Immortalized human astrocytes were stably infected with wild-type *EGFR* or the indicated *EGFR* missense mutants. Shown are total phosphotyrosine (PY), Y1068-EGFR, total EGFR, and PI3K p85 (loading control) immunoblots of whole cell lysates from cells following 12 h of serum starvation. The solid arrow at the PY position represents tyrosine-phosphorylated EGFR, and the interrupted arrows indicated other differentially tyrosine-phosphorylated proteins. The inset shows an anti-EGFR immunoblot of parental astrocytes (far-left lane) and stable astrocyte subclones (designated in remaining five lanes) growing in full serum. (C) Basal receptor phosphorylation and EGF-responsiveness of wild-type EGFR and four different EGFR ectodomain mutants stably expressed in Ba/F3 murine hematopoietic cells. Shown are immunoblots of stable Ba/F3 subclones after 12 h of serum starvation (− EGF) and 15 min following EGF-induction (0.5 or 5 ng/ml EGF).

Signal transduction through EGFR is determined by its basal catalytic activity, receptor activation by ligand, and signal termination through intracellular compartmentalization of the receptor-ligand complex, receptor dephosphorylation, and degradation [27]. To explore the biochemical basis for the gain of function observed with EGFR ectodomain mutants, we first examined the basal catalytic activity of A289V-EGFR in transiently transfected 293T cells using EGFR autophosphorylation as a readout for receptor activation. EGFR autophosphorylation was determined by measuring the total phosphotyrosine content of the immunoprecipitated receptor (Figure 3A, left blot) and by immunoblotting of whole cell lysates with several phosphosite-specific anti-EGFR antibodies (Figure 3A, right blot). Compared to wild-type EGFR, the ectodomain mutant A289V-EGFR showed a marked increase in receptor autophosphorylation in the absence of ligand or serum. We subsequently examined a more extensive panel of EGFR missense mutants (T263P, A289V, G598V, L861Q) in immortalized human astrocytes stably transduced with these receptors. Compared to astrocytes overexpressing wild-type EGFR, sublines expressing EGFR missense mutants showed an increased phosphotyrosine content of EGFR and several other unidentified proteins under serum-free conditions (Figure 3B).

We also expressed selected EGFR mutants (R108K, T263P, A289V, G598V, L861Q) in murine hematopoietic cells (Ba/F3 cells) which do not express any EGFR family members [14] but otherwise retain functional properties of the EGF-signaling pathway [28–30]. Consistent with our findings in 293T cells and astrocytes, all examined EGFR ectodomain mutants showed increased tyrosine phosphorylation under serum-starved conditions and were responsive to exogenous EGF (Figure 3C). We also noted that EGF stimulation led to a more pronounced drop of EGFR levels in Ba/F3 cells expressing wild-type EGFR than in subclones expressing EGFR ectodomain mutants (Figure 3C), reminiscent of the impaired ligand-induced receptor downregulation reported for selected EGFR kinase domain mutants [31].

### Sensitivity of EGFR Ectodomain Mutants to EGFR Kinase Inhibitors

**Figure 4:**
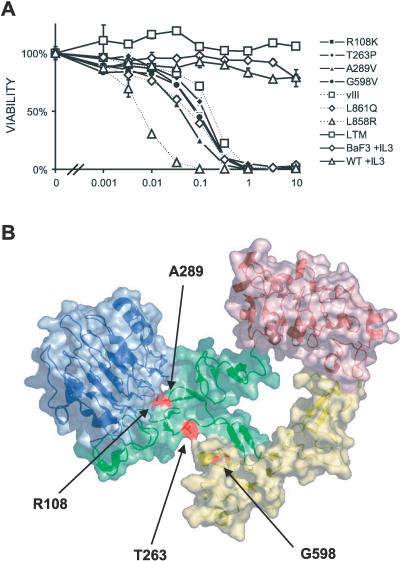
*EGFR* Missense Mutations Sensitize Cells to EGFR Kinase Inhibitors (A) Effect of increasing concentrations of the EGFR inhibitor erlotinib (0–10 μM) on the viability of IL-3 independent Ba/F3 subclones expressing EGFR ectodomain mutants (R108K, T263P, A289V, G598V, and EGFRvIII), the EGFR kinase domain mutants (L858R and L861Q), or the erlotinib-resistant EGFR double mutant L858R-T790M (LTM). Parental Ba/F3 cells and Ba/F3 cells expressing wild-type EGFR are not IL-3 independent and were included as controls. Viability (a mean percent of control ± standard deviation) was determined after exposure to erlotinib for 48 h. (B) Oncogenic EGFR ectodomain mutations map to interdomain interfaces. Shown are ribbon and surface diagrams of the EGFR [46] with sites of amino acid substitutions highlighted. Blue, domain I; green, domain II; red, domain III; and yellow, domain IV. Sites of the most prevalent amino acid substitutions are shown in red. Images were created with PyMOL (http://pymol.sourceforge.net/). P596 is not visible in this view.

The presence of identical missense mutations in multiple patient samples and their oncogenicity in standard transformation assays suggest that these mutants play a role in gliomagenesis. It also raises the question whether these mutations might sensitize transformed cells to EGFR kinase inhibitors. Ba/F3 cells provide a unique model system to examine kinase inhibitor sensitivity [32–35] because stable expression of oncogenic kinases in these cells can relieve them from their intrinsic dependence on IL-3 for survival [15,36]. As expected from our results in NIH-3T3 cells, expression of the tested EGFR missense mutants but not wild-type EGFR was able to relieve Ba/F3 cells from IL-3 dependence. Addition of the EGFR kinase inhibitor erlotinib to the media had little or no effect on the viability of parental Ba/F3 cells growing in the presence of IL-3 or on Ba/F3 cells expressing the drug-resistant EGFR double-mutant L858R/T790M-EGFR. However, erlotinib did induce dose-dependent cell death in Ba/F3 subclones expressing the EGFR ectodomain mutants (missense and vIII truncation) or EGFR kinase domain mutants (L858R and L861Q) (Figure 4A). Of note, erlotinib-induced cell death of Ba/F3 cells expressing EGFR ectodomain mutants occurred at IC-50 values of 50–150 nM, drug concentrations that are well below the concentrations achieved in human plasma [37]. These data suggest that EGFR missense mutants sensitize transformed cells to EGFR kinase inhibitors similar to EGFRvIII or lung cancer-related kinase domain mutants, both of which have been associated with clinical responses to EGFR kinase inhibitor therapy [9,19,38,39].

We recently reported the results of a glioblastoma clinical trial with EGFR kinase inhibitors which associated clinical responses to the coexpression of EGFRvIII and PTEN [9]. To investigate whether clinical responses might also be linked to the presence of *EGFR* ectodomain mutations, we reexamined all available tumor DNA samples from this clinical trial. We identified the ectodomain mutant R108K-*EGFR* in 14% (1/7) gliomas that responded to erlotinib. This tumor, however, also expressed *EGFRvIII,* raising the possibility of independent clones arising from a common progenitor with *EGFR* amplification. We also identified the R108K *EGFR* mutation in 7% (1/5) gliomas that failed EGFR kinase inhibitor therapy, but loss of PTEN in this tumor provides a potential explanation for treatment failure (Table S3). Larger clinical trials are required to ascertain the contribution of EGFR missense mutants to EGFR kinase inhibitor response in glioblastoma.

## Discussion

We have identified novel oncogenic missense mutations in the ectodomain of *EGFR* in glioma. The association of these mutations with increased *EGFR* gene dosage raises the question of whether similar ectodomain missense mutations might exist in other malignancies with *EGFR* amplification or polysomy of Chromosome 7. More broadly, our results suggest that ectodomain missense mutations in other tyrosine kinase genes may be transforming events in multiple cancers, arguing for an extension of current kinase gene resequencing efforts beyond the kinase domains [40,41].

The ligand-independent basal phosphorylation of the EGFR missense mutants in our study is consistent with their ability to confer NIH-3T3 cells with the ability to grow in soft agar in the absence of exogenous EGF. Whether all EGFR ectodomain mutants share a common mechanism of oncogenic receptor conversion warrants further study. A common mechanism is suggested by the structural observation fact that many of the resulting amino acid substitutions map to interdomain interfaces. R108K and A289V/D/T occur at the domain I/II interface, P569L and G598V occur at the domain II/IV contact, and T263P occurs in domain II just before the extended loop that contacts domain IV (Figure 4B). Differences in constitutive receptor activity (G598V>A289V>T263P), on the other hand, point toward alternative mechanisms of oncogenic receptor conversion.

Three of the *EGFR* missense mutations (encoding P596L, G598V, and A289V) were previously observed in smaller cohorts of glioblastoma tumors [24,42]. The identification of additional ectodomain mutations in our study might have been facilitated by the large number of tumors, near-complete coverage of the *EGFR* coding sequence, and use of MALDI-TOF mass spectrometry genotyping in addition to Sanger sequencing. Since most of the patients in our study were of Northern European descent, we were unable to establish whether the prevalence of *EGFR* ectodomain mutations in glioblastoma might be affected by ethnicity as has been shown for *EGFR* kinase domain mutations. The distribution of *EGFR* missense mutations in glioblastoma (largely extracellular) and lung cancer (exclusively kinase domain) suggests fundamental differences in oncogenic EGFR signaling between these two tumor types. Importantly, however, both classes of mutants—as well as EGFRvIII—appear to sensitize transformed cells to EGFR kinase inhibitors in a preclinical model system that has been predictive of clinical responses [33,43]. Based on the experience with kinase inhibitors for chronic myeloid leukemia [44], the development of sensitive methodologies to monitor the EGFR pool before and during therapy will constitute an important step in advancing the current use of EGFR kinase inhibitors for cancer.

## Supporting Information

Figure S1 Protein Sequence Alignment for EGFR Missense Mutations Alignment (ClustalW) of the human EGFR protein sequences ERBB2, ERBB3, ERBB4, and the EGFR protein sequence of *Mus musculus, Rattus norvegicus, Sus scrofa, Danio rerio,* and Drosophila melanogaster and *Xiphophorus* for the residues affected by missense mutations. (29 KB PDF)

Table S1 PCR and Extension Primers for MALDI-TOF Mass Spectrometry Genotyping of 24 SNPs (18 KB XLS)

Table S2 PCR and Extension Primers for EGFR MALDI-TOF Mass Spectrometry Genotyping (15 KB XLS)

Table S3 Ectodomain Mutations in Glioblastomas and Response to EGFR Kinase Inhibitor Therapy Patients were classified as responders (patients 1–7) or nonresponders (patients 8–26) based on radiographic criteria [9].
(16 KB XLS)

### Accession Numbers

The GenBank (http://www.ncbi.nlm.nih.gov) accession numbers of the proteins discussed in this paper are human EGFR protein (NM_005228), ERBB2 26 (NM_004448), ERBB3 (NM_001982), ERBB4 (NM_005235); and the EGFR proteins of Mus musculus (NM_207655), Rattus norvegicus (NM_031507), Sus scrofa (NM_214007), Danio rerio (NM_194424), Drosophila melanogaster (NM_057410), and *Xiphophorus* (X56319)

## Abbreviations

EGF: epidermal growth factor
EGFR: epidermal growth factor receptor
FISH: fluorescence in situ hybridization
IL-3: interleukin-3
MALDI-TOF: matrix-assisted laser desorption/ionization time-of-flight
SNP: single-nucleotide polymorphism
