# Supplementary material for: Epidermal Growth Factor Receptor Activation in Glioblastoma through Novel Missense Mutations in the Extracellular Domain
Source: PLoS Med. 2006 Dec 19;3(12):e485. doi: 10.1371/journal.pmed.0030485 (PMC1702556; doi:10.1371/journal.pmed.0030485)
Supplement: Supplementary file 1 [file pmed.0030485.sg001.pdf]

Supplementary Figure S1

D46N G63R

```
HOMO SAPIENS EGFR            KVCQGTSNKLTQLGTFEDHFLSLQRMFNNCEVVLGN-LEITYVQ-RNYDL 76
HOMO SAPIENS HER2 (ERBB2)    QVCTGTDMKLRLPASPETHLDMLRHLYQGCQVVQGN-LELTYLP-TNASL 71
HOMO SAPIENS HER3 (ERBB3)    AVCPGTLNGLSVTGDAENQYQTLYKLYERCEVVMGN-LEIVLTG-HNADL 74
HOMO SAPIENS ERBB4           SVCAGTENKLSSLSDLEQQYRALRKYYENCEVVMGN-LEITSIE-HNRDL 74
MUS MUSCULUS                 KVCQGTSNRLTQLGTFEDHFLSLQRMYNNCEVVLGN-LEITYVQ-RNYDL 76
RATTUS NORVEGICUS            KVCQGTSNRLTQLGTFEDHFLSLQRMFNNCEVVLGN-LEITYVQ-RNYDL 76
SUS SCROFA                   KVCQGTSNKLTQLGTFEDHFLSLQRMFNNCEVVLGN-LEITYMQ-NSYNL 76
DANIO RERIO                  KVCQGANNKLTLLGTVEDHYQVLLRMYRNCTVVLEN-LEITHIT-EKYDL 73
DROSOPHILA MELANOGASTER      KVCIGTKSRLSVPSNKEHHYRNLRDRYTNCTYVDGN-LELTWLPNENLDL 99
XIPHOPHORUS (XMRK)           KVCQGTSNQMTML---DNHYLKMKKMYSGCNVVLENTLEITYTQ-ENQDL 73
                              ** *:   :       : :   :   :   *   *   * **:.     . .*
```

R324L E330K

```
HOMO SAPIENS EGFR            YEME-EDGVRKCKKCEGPCRKVCNGIGIGEFKDSLSINATNIKHFKNCTS 364
HOMO SAPIENS HER2 (ERBB2)    QEVTAEDGTQRCEKCSKPCARVCYGLGMEHLREVRAVTSANIQEFAGCKK 369
HOMO SAPIENS HER3 (ERBB3)    MEVD-KNGLKMCEPCGGLCPKACEGTGSGSRF--QTVDSSNIDGFVNCTK 356
HOMO SAPIENS ERBB4           MEVE-ENGIKMCKPCTDICPKACDGIGTGSLMSAQTVDSSNIDKFINCTK 361
MUS MUSCULUS                 YEVE-EDGIRKCKKCDGPCRKVCNGIGIGEFKDTLSINATNIKHFKYCTA 364
RATTUS NORVEGICUS            YEVE-EDGVSKCKKCDGPCRKVCNGIGIGEFKDTLSINATNIKHFKYCTA 364
SUS SCROFA                   YEVE-EDGVRKCKKCDGPCGKVCNGIGIGEFKDTLSINATNIKHFRNCTS 364
DANIO RERIO                  YEVD-EGGVRKCKRCEGLCPKVCNGLGMGPLANVLSINATNIDSFENCTK 362
DROSOPHILA MELANOGASTER      MDKG-----GECVPCNGPCPKTCPGVT--------VLHAGNIDSFRNCTV 373
XIPHOPHORUS (XMRK)           LEVD-ENGKRSCKPCDGVCPKVCDGIGIGSLSNTIAVNSTNIGSFSNCTK 366
                              :             *   *    *  :.* *            : : **   *   *.
```

R108K

```
HOMO SAPIENS EGFR            SFLKTIQEVAGYVLIALNTVERIPLENLQIIRGNMYYENSYALAVLSNYD 126
HOMO SAPIENS HER2 (ERBB2)    SFLQDIQEVQGYVLIAHNQVRQVPLQRLRIVRGTQLFEDNYALAVLDNGD 121
HOMO SAPIENS HER3 (ERBB3)    SFLQWIREVTGYVLVAMNEFSTLPLPNLRVVRGTQVYDGKFAIFVMLNYN 124
HOMO SAPIENS ERBB4           SFLRSVREVTGYVLVALNQFRYLPLENLRIIRGTKLYEDRYALAIFLNYR 124
MUS MUSCULUS                 SFLKTIQEVAGYVLIALNTVERIPLENLQIIRGNALYENTYALAILSNYG 126
RATTUS NORVEGICUS            SFLKTIQEVAGYVLIALNTVERIPLENLQIIRGNALYENTYALAVLSNYG 126
SUS SCROFA                   SFLKTIQEVAGYVLIALNTVEKIPLENLQIIRGNVLYENTHALAVLSNYG 126
DANIO RERIO                  SFLKSIQEVGGYVLIAVNTVSKIPLENLRIIRGHSLYEDKFALAVLVNFN 123
DROSOPHILA MELANOGASTER      SFLDNIREVTGYILISHVDVKKVVFPKLQIIRGRTLFSLSVEEEKYALFV 149
XIPHOPHORUS (XMRK)           SFLQSIQEVGGYVLIAMNEVSTIPLVNLRLIRGQNLYEGNFTLLVMSNYQ 123
                             ***  ::** **:*::   .  : :  .*:::**   :.
```

P596L

```
HOMO SAPIENS EGFR            CIQCHPECLPQAM-NITCTGRGPDNCIQCAHYIDGPHCVKTCP------- 596
HOMO SAPIENS HER2 (ERBB2)    CLPCHPECQPQNG-SVTCFGPEADQCVACAHYKDPPFCVARCP------- 601
HOMO SAPIENS HER3 (ERBB3)    CFSCHPECQPMEG-TATCNGSGSDTCAQCAHFRDGPHCVSSCP------- 590
HOMO SAPIENS ERBB4           CVECDPQCEKMEDGLLTCHGPGPDNCTKCSHFKDGPNCVEKCP------- 594
MUS MUSCULUS                 CIQCHPECLPQAM-NITCTGRGPDNCIQCAHYIDGPHCVKTCP------- 596
RATTUS NORVEGICUS            CIQCHPECLPQTM-NITCTGRGPDNCIKCAHYVDGPHCVKTCP------- 596
SUS SCROFA                   CVQCHPECLPQAK-NVTCMGRGPDSCVRCAHYIDGPHCVKTCP------- 596
DANIO RERIO                  CMECDPECLLMNE-TQTCNGPGPDKCTVCANYKDGPHCVHRCP------- 594
DROSOPHILA MELANOGASTER      CKICHPECR-------TCNGAGADHCQECVHVRDGQHCVSECPKNKYNDR 613
XIPHOPHORUS (XMRK)           CVQCHQECLVQTD-SLTCYGPGPANCSKCAHFQDGPQCIPRCP------- 591
                             *  *. :*        ** *   .  *   *  :  *    *:  **
```

T263P

```
HOMO SAPIENS EGFR            QQCSG-RCRGKSPSDCCHNQCAAGCTGPRESDCLVCRKFRDEATCKDTCP 265
HOMO SAPIENS HER2 (ERBB2)    GGCA--RCKGPLPTDCCHEQCAAGCTGPKHSDCLACLHFNHSGICELHCP 269
HOMO SAPIENS HER3 (ERBB3)    PQCNG-HCFGPNPNQCCHDECAGGCSGPQDTDCFACRHFNDSGACVPRCP 260
HOMO SAPIENS ERBB4           EQCDG-RCYGPYVSDCCHRECAGGCSGPKDTDCFACMNFNDSGACVTQCP 263
MUS MUSCULUS                 QQCSH-RCRGRSPSDCCHNQCAAGCTGPRESDCLVCQKFQDEATCKDTCP 265
RATTUS NORVEGICUS            QQCSR-RCRGRSPSDCCHNQCAAGCTGPRESDCLVCHRFRDEATCKDTCP 265
SUS SCROFA                   QQCSG-RCRGRSPSDCCHNQCAAGCTGPRESDCLVCRRFRDEATCKDTCP 265
DANIO RERIO                  EQCSG-RCKGPRPIDCCNEHCAAGCTGPRPTDCLACKDFQDEGTCKDACP 263
DROSOPHILA MELANOGASTER      PQCAGGRCYGPKPRECCHLFCAGGCTGPTQKDCIACKNFFDEGVCKEECP 287
XIPHOPHORUS (XMRK)           EQCNR-RCRGPKPIDCCNEHCAGGCTGPRATDCLACRDFNDDGTCKDTCP 268
                               *   :* *    :**:  **.**:**  .**:.*  *  ... *   **
```

G598V

```
HOMO SAPIENS EGFR            ----------AGVMGENNTL-V---------------------------- 607
HOMO SAPIENS HER2 (ERBB2)    ----------SGVKPDLSYMPI---------------------------- 613
HOMO SAPIENS HER3 (ERBB3)    ----------HGVLGAKGPI------------------------------ 600
HOMO SAPIENS ERBB4           ----------DGLQGANSFI------------------------------ 604
MUS MUSCULUS                 ----------AGIMGENNTL-V---------------------------- 607
RATTUS NORVEGICUS            ----------SGIMGENNTL-V---------------------------- 607
SUS SCROFA                   ----------AGIAGENSTL-I---------------------------- 607
DANIO RERIO                  ----------QGVPGEKDTL-I---------------------------- 605
DROSOPHILA MELANOGASTER      GVCRECHATCDGCTGPKDTIGIGACTTCNLAIINNDATVKRCLLKDDKCP 663
XIPHOPHORUS (XMRK)           ----------HGMLGDGDTL-I---------------------------- 602
                                        *      . :
```

A289D/T/V

```
HOMO SAPIENS EGFR            PLMLYNPTTYQMDVNPEGKYSFGATCVKKCPRNYVVTDHGSCVRACGADS 315
HOMO SAPIENS HER2 (ERBB2)    ALVTYNTDTFESMPNPEGRYTFGASCVTACPYNYLSTDVGSCTLVCPLHN 319
HOMO SAPIENS HER3 (ERBB3)    QPLVYNKLTFQLEPNPHTKYQYGGVCVASCPHNFVVDQT-SCVRACPPDK 309
HOMO SAPIENS ERBB4           QTFVYNPTTFQLEHNFNAKYTYGAFCVKKCPHNFVVDSS-SCVRACPSSK 312
MUS MUSCULUS                 PLMLYNPTTYQMDVNPEGKYSFGATCVKKCPRNYVVTDHGSCVRACGPDY 315
RATTUS NORVEGICUS            PLMLYNPTTYQMDVNPEGKYSFGATCVKKCPRNYVVTDHGSCVRACGPDY 315
SUS SCROFA                   PLMLYNPTTYQMDVNPLGKYSFGATCVKKCPRNYVVTDHGSCVRACSSDS 315
DANIO RERIO                  RLMLYDPNTHQLAPNPYGKYSFGATCIKTCPHNYVVTDHGACVRTCSPGT 313
DROSOPHILA MELANOGASTER      PMRKYNPTTYVLETNPEGKYAYGATCVKECPG-HLLRDNGACVRSCPQDK 336
XIPHOPHORUS (XMRK)           PPKIYDIVSHQVVDNPNIKYTFGAACVKECPSNYVVTEG-ACVRSCSAGM 317
                                 *:  :.     *    :* :*. *:  **   .:  .   :*.  *
```

L861Q

```
HOMO SAPIENS EGFR            RDLAARNVLVKTPQHVKITDFGLAKLLGAEEKEYHAEGGKVPIKWMALES 885
HOMO SAPIENS HER2 (ERBB2)    RDLAARNVLVKSPNHVKITDFGLARLLDIDETEYHADGGKVPIKWMALES 893
HOMO SAPIENS HER3 (ERBB3)    RNLAARNVLLKSPSQVQVADFGVADLLPPDDKQLLYSEAKTPIKWMALES 882
HOMO SAPIENS ERBB4           RDLAARNVLVKSPNHVKITDFGLARLLEGDEKEYNADGGKMPIKWMALEC 891
MUS MUSCULUS                 RDLAARNVLVKTPQHVKITDFGLAKLLGAEEKEYHAEGGKVPIKWMALES 887
RATTUS NORVEGICUS            RDLAARNVLVKTPQHVKITDFGLAKLLGAEEKEYHAEGGKVPIKWMALES 886
SUS SCROFA                   RDLAARNVLVKTPQHVKITDFGLAKLLGAEEKEYHAEGGKVPIKWLALES 885
DANIO RERIO                  RDLAARNVLVKTPQHVKITDFGLAKLLNADEKEYHADGGKVPIKWMALES 884
DROSOPHILA MELANOGASTER      RDLAARNVLVQTPSLVKITDFGLAKLLSSDSNEYKAAGGKMPIKWLALEC 1062
XIPHOPHORUS (XMRK)           RDLAARNVLLKNPNHVKITDFGLSKLLTADEKEYQADGGKVPIKWMALES 880
                             *:*******::.*.  *:::***:: **  :..:     .* ****:***.
```
